# Biomechanical Evaluation of Preoperative Rehabilitation in Patients of Anterior Cruciate Ligament Injury

**DOI:** 10.1111/os.12607

**Published:** 2020-03-08

**Authors:** Wei Li, Zhongli Li, Shuyan Qie, Ji Li, Jia‐ning Xi, Wei‐jun Gong, Yue Zhao, Xue‐mei Chen

**Affiliations:** ^1^ Department of Orthopedics General Hospital of PLA Beijing China; ^2^ Department of Rehabilitation Beijing Rehabilitation Hospital, Capital Medical University Beijing China; ^3^ Department of Health Management Beijing Rehabilitation Hospital, Capital Medical University Beijing China

**Keywords:** Anterior cruciate ligament (ACL) injury, Gait analysis, Surface electromyography, Proprioception test, Rehabilitation

## Abstract

**Objectives:**

To investigate the biomechanical characteristics of patients with anterior cruciate ligament (ACL) injury by gait analysis, surface electromyography (SEMG), and proprioception test, and provide rehabilitation suggestions according to the results.

**Methods:**

In this retrospective cohort study, 90 adults with unilateral ACL injury, ranging in age from 19 to 45 years (66 men and 24 women, average age: 30.03 ± 7.91) were recruited for this study form May 2018 to July 2019. They were divided into three groups according to the time after the injury: group A (3‐week to 1.5‐month), group B (1.5‐month to 1 year), and group C (more than 1 year). The SEMG signals were collected from the bilateral rectus femoris (RF), vastus medialis (VM), and vastus lateralis (VL) and the root mean square (RMS) were used to assess muscular activity. SEMG were used to analyze muscles function, gait analysis was used to evaluate the walking stability, balance and location assessment were used to analyze the proprioception.

**Results:**

Through the comparison between bilateral limbs, all muscles strength shown decreased (RF: 239.94 ± 129.70 *vs* 364.81 ± 148.98, *P* = 0.001; VM: 298.88 ± 175.41 *vs* 515.79 ± 272.49, *P* = 0.001; VL:389.54 ± 157.97 *vs* 594.28 ± 220.31, *P* < 0.001) and the division of proprioception became larger (tandem position: 7.79 ± 1.57 *vs* 6.33 ± 1.49, *P* = 0.001; stance with one foot: 8.13 ± 0.84 *vs* 7.1 ± 0.57, *P* = 0.003; variance of 30°: 6.96 ± 3.15 *vs* 4.45 ± 1.67, *P* = 0.03; variance of 60°: 4.64 ± 3.38 *vs* 2.75 ± 1.98, *P* = 0.044) in the injured side when compared to the non‐injured and 26 gait parameters were shown difference in group A. In group B, the muscle strength of VL shown decreased (VL: 381.23 ± 142.07 *vs* 603.9 ± 192.72, *P* < 0.001) and the division of location of 30° became larger (7.62 ± 4.98 *vs* 4.33 ± 3.24, *P* = 0.028) in the injured side when compared to the non‐injured side and there were eight gait parameters that showed differences. In group C, the muscle strength and proprioception showed no differences and only 16 gait parameters showed differences between the bilateral limbs.

**Conclusion:**

The results proved the deterioration of proprioception in 30° of injured side will not recover and non‐injury side and will become worse after 1 year from the injury; among the VL, VM, and RF, the recovery rate of VL is the slowest and bilateral straight leg raising (SLR) (30°) is the best way to train it; the gait stability will be worse after 1 year from the injury. Therefore, we suggest that the training for proprioception in 30° and VL are important for the rehabilitation, and the ACL reconstruction should be performed within 1 year.

## Introduction

The anterior cruciate ligament (ACL) is one of the most important ligaments in the knee, and experiences approximately 200,000 isolated injuries annually^1^. This situation is even worse in the army; a study by Pietrosimone showed that ACL injury occurrence was approximately four to five times higher among the military than the general population^2^. Anterior cruciate ligament reconstruction (ACLR) has been recommended as the best procedure to rebuild the function of ACL^3^ and this procedure could achieve good results regardless of the grafts material hamstrings^4^ or patellar tendon^5^. However, the restoration of athletic ability was not satisfied. Myklebust and Bahr^6^ reported only 58% of the participants who underwent reconstruction operation were able to return to their preinjury sports participation and 44% were still under the high level of preinjury. Therefore, how to improve the performance of the patients with ACL injury and reduce the injury risk is an urgent problem to be solved.

Pre‐surgery rehabilitation of ACL injuries was stated as playing an important role for the functional recovery after operation^7^. Shaarani^8^ proved a 6‐week preoperative rehabilitation program could improve the outcomes at 12 weeks after surgery. Failla *et al*.^9^ reported that delaying surgery until completing a whole pre‐operative rehabilitation program could improve functional knee scores by 12% to 15% when compared with a group that did not engage in a pre‐rehabilitation program. Shelbourne *et al*.^10^ also reported that the development of arthrofibrosis was reduced from 17% in the immediate surgery group to 0% when delaying surgery until the knee joint calms down following ACL injury. Therefore, pre‐operation rehabilitation could possibly serve as an effective way to improve the recovery to a high level after ACLR surgery. Gait analysis is a precise way to provide clinicians with kinematics and kinetics data for dynamic function and gait pattern. Therefore, it can be an efficient way to detect the gait characteristic of patients. Lee *et al*.^11^ reported the decrease of velocity, stride length, and cadence in patients with nerve disorder. Morag *et al*.^12^ also proposed the kinetic data that was calculated in gait analysis is a valuable indicator which could indicate the agonist and antagonist function. Surface electromyography (SEMG) is also another method to evaluate muscle function. More and more experts believe that a precise assessment of the neuromuscular function should include both gait analysis and SEMG, as the combined method shows higher accuracy to assess the physiological and functional status of peripheral nervous systems rather than simply anatomical and structural evaluation^13^. Di Nardo *et al.* 14 devised a method to investigated patients with lumbar spinal stenosis^15^, hemiplegic cerebral palsy, and stroke^16^.

Nowadays, the rehabilitation program for the patients with ACL injury is mainly based on the time after injury or the experience of rehabilitation therapists, and few based on the gait analysis, SEMG, and other sports biomechanics research methods^17^. We hypothesized that the patients with ACL injury will show different characteristics in different stages after injury. Therefore, the purpose of this study is to evaluate the biomechanics characteristics of patients with ACL injury at different stages using gait analysis and SEMG; additionally, we sought to evaluate the characteristics of proprioception of patients with ACL injury at different stages; finally, we determined to provide a rehabilitation program with statistical evidence according to biomechanics results.

## Patients and Methods

### *Patient Data*

Ninety adults with unilateral ACL injury, ranging in age from 19 to 45 years (66 men and 24 women, average age: 30.03 ± 7.91 years; height: 172.69 ± 8.78 cm; weight: 72.01 ± 13.48 kg) were recruited for this study form May 2018 to July 2019, and they were divided into three groups according to the time after the injury: group A (3‐weeks to1.5‐months), group B (1.5‐months to 1 year), and group C (more than 1 year) 18.

The inclusion criteria were: (i) patients with unilateral ACL injury and age >18 years; (ii) patients who received no rehabilitation treatment and training in hospital; (iii) the main evaluation indicators included the root mean square (RMS) of SEMG, the spatiotemporal parameters of gait as single support (ms), double dupport (ms), etc., the center of pressure (COP) velocity (cm/s) and the discrepancy of angle; (iv) the impact of the time after injury; and (v) a retrospective cohort study. The exclusion criteria were: (i) patients with knee disorders, anatomical abnormalities; and (ii) history of surgery in lumbar and lower limbs. The experiment was approved by the Beijing Rehabilitation Hospital. A signed consent form was obtained from each subject before any testing was performed.

### *SEMG Measurement*

**Figure 1:**
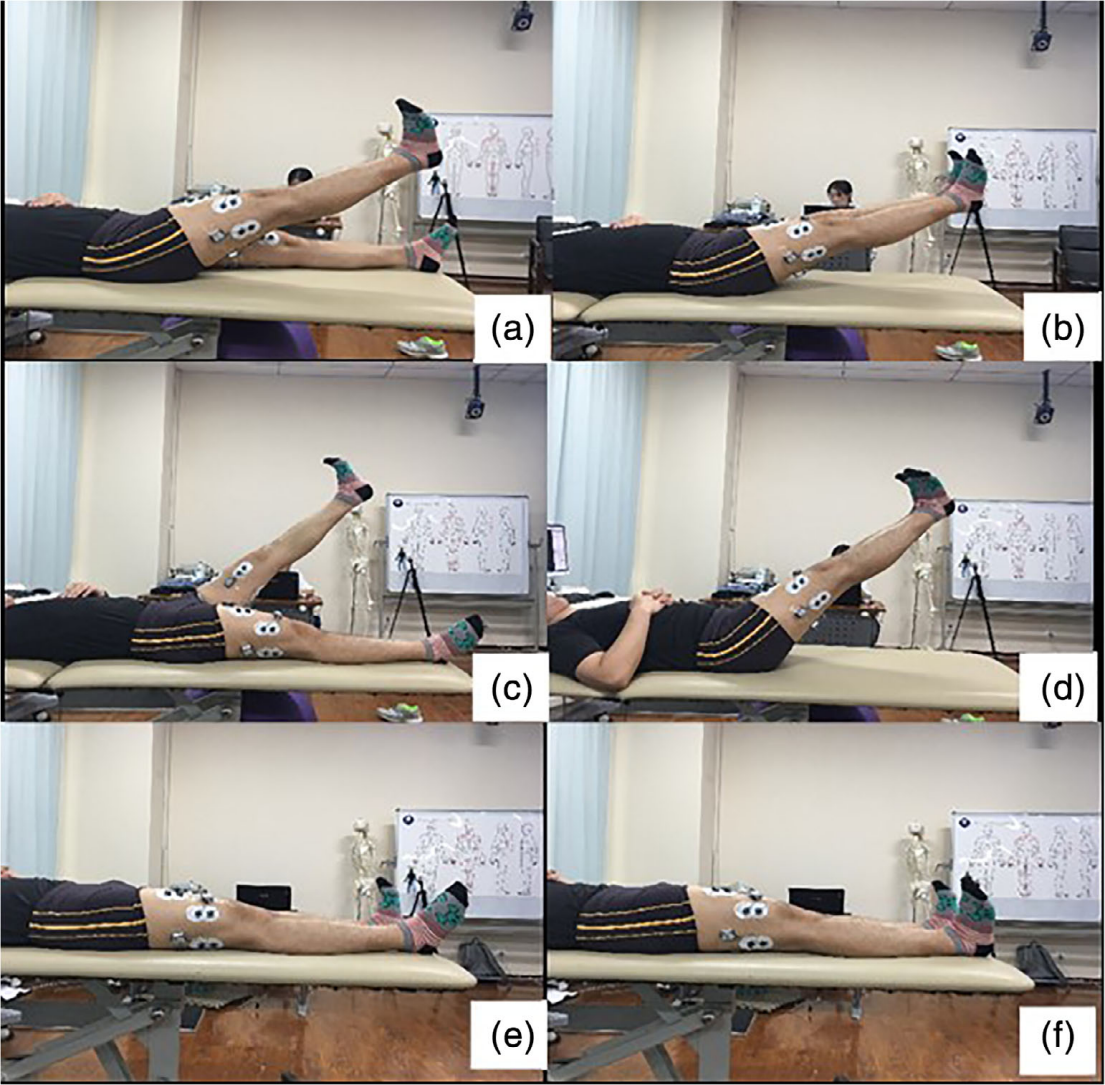
The tasks performed during experiment include: Single‐SLR (30°) (A), Both‐SLR (30°) (B), Single SLR (60°) (C), Both SLR(60°) (D), Single‐ankle dorsiflexion (E) and Both‐ankle dorsiflexion (F).

SEMG was measured using the Noraxon wireless dynamic electromyography tester (Noraxon, USA Inc. Scottsdale, AZ). Electrodes were pasted on patients' skin after scraping, abrading, and alcohol cleaning. The SEMG signals were collected from the bilateral rectus femoris (RF), vastus medialis (VM), and vastus lateralis (VL), and were sampled at 1200 Hz with a band pass filter of 20–500 Hz. The RMS were used to assess muscular activity. The tasks performed during the experiment include: single straight leg raising (SLR) (30°), both‐SLR (30°), single SLR (60°), both SLR (60°), single‐ankle dorsiflexion and both‐ankle dorsiflexion (Fig. 1).

### *Gait Measurement*

The Intelligent Device for Energy Expenditure and Activity (IDEEA, MiniSun, LLC, Fresno, CA, USA), which is equipped with accelerometer and gyroscopes, was applied to monitor physical activity and measure gait parameters. The IDEEA has been used widely in clinical stud^19^. The patients walked at a comfortable speed on a 50‐meter runway and the tasks for the test included: walking, fast walking, inverted walking, serpentine walk, walking with dual task, upstairs, downstairs, and normal walking after warm‐up. The parameters that were used in the analysis included: single support (ms), double support (ms), SLS/DLS (%), swing duration (ms), step duration (ms), cycle duration (sec), pulling accel (G), swing power (G), ground impact (G), foot fall (G), push off (G), speed (m/min), cadence (steps/min), step length (meters), and stride length (meters).

### *Proprioception Measurement*

This part contains balance assessment and location assessment. In the balance assessment study, all subjects completed the standardized balance test with the Stability and Balance Learning Environment System (DynSTABLE, Motek medical Corporation, Amsterdam, Netherlands). The system offers real‐time integration of a translational balance platform, a force plate, motion capture system, and virtual reality environments. The outcome variables were measured in the following four trials: (i) stance with eyes open (EO); (ii) stance with eyes closed (EC); (iii) stance with full tandem position (with one heel placed directly in front of the other foot); and (iv) single leg stance. The COP velocity of the foot was calculated from the components of forces of the registered plate response, which were separately analyzed in then medio‐lateral (ML) and anterior–posterior (AP) planes. Each successive measurement using the force plate lasted for 10 s and postural sway was calculated.

In the angular positioning study, all subjects completed the standardized balance test with the Biodex System four isokinetic dynamometer (Biodex Medical Systems, Shirley, New York, USA). This system is a contemporary isokinetic dynamometer with an electrically controlled servomechanism used in both clinical and research settings. Biodex dynamometers have been proved to be reliable and valid instruments for the measurement of human function^20^. Discrepancy was calculated as the difference between the criterion angle and actual achieved angle recorded by the dynamometer's electrogoniometer. The outcome variables were measured in the following four trials: (i) measurement of 30° (the movement start from 90° and let the subject brake at the degree of 30° according to their sensation); and (ii) measurement of 60° (the movement start from 90° and let subjects brake at the degree of 60°).

### *Outcome Measures*

#### *The SEMG Evaluation*

The RMS value of SEMG is a parameter that reflects the muscle function and it is used in this study to evaluated the maximum voluntary contraction (MVC) of muscle. They were measured by the SEMG – the larger the value, the more muscle fibers are collected and reflect greater muscle strength.

#### *The Gait Measurement*

The single support (ms), double support (ms), etc., were the spatiotemporal parameters of gait analysis and were used to evaluate the walking ability of patients. They were measured by the IDEEA, where the larger difference in parameters between bilateral sides means the worse the walking ability.

#### *The Proprioception Measurement*

This measurement contains balance assessment and location assessment, and they are parameters that reflect the change of proprioception. The balance ability was evaluated by the COP velocity of the foot and is recorded as cm/s. The location was evaluated by the discrepancy between the criterion angle and actual achieved angle. The larger the value, the worse the proprioception.

### *Statistical Methods*

All the data was analyzed in SPSS software (Version 19.0, IL, USA). Shapiro–Wilk normality test was performed to examine the normality distribution of the data and t‐test was applied during the comparison between the both sides. One‐way chi‐square analysis for selective two‐to‐two comparisons was carried out among multiple groups of data. Values of *P* < 0.05 were considered significantly different.

## Results

All 90 Chinese soldiers completed the tests. The basic information of all groups were shown in Table 1. The circumference of thigh in 10 cm and 15 cm increments shows a decrease of the injured side when compared to the non‐injured side in both group A and group B (Table 2).

**Table 1:** The general characteristics of different groups

Indexes	A	B	C
N	30	30	30
Age(yr)	29.83 ± 7.87	30.5 ± 6.29	29.75 ± 9.26
Weight(kg)	173.83 ± 9.95	172.92 ± 5.79	171.33 ± 9.77
Height(m)	72.42 ± 16.08	71.29 ± 8.97	72.33 ± 14.34
Female	8	10	6
Male	22	20	24

**Table 2:** The circumference of thigh

		Group A			Group B		Group C		
	symptomatic side	asymptomatic side	*P*‐value	symptomatic side	asymptomatic side	*P*‐value	symptomatic side	asymptomatic side	*P*‐value
10cm	47.90 ± 4.69	50.7 ± 6.28	0.027*	48.48 ± 4.83	51.4 ± 6.18	0.018*	48.50 ± 0.71	49.75 ± 1.06	0.561
15cm	51.42 ± 5.27	53.36 ± 5.39	0.014*	51.17 ± 4.90	54.38 ± 4.54	0.014*	53.70 ± 0.42	54.20 ± 1.13	0.472

* There are significant differences.

### *SEMG Measurement Results*

The maximum voluntary contraction (MVC) of bilateral lower limbs were significantly different among groups. In group A, all of the muscles (including RF, VM, and VL) decreased significantly in the injured side (RF: 239.94 ± 129.70 *vs* 364.81 ± 148.98, *P* = 0.001; VM: 298.88 ± 175.41 *vs* 515.79 ± 272.49, *P* = 0.001; VL: 389.54 ± 157.97 *vs* 594.28 ± 220.31, *P* < 0.001). In group B, only VL decreased in the injured side when compared to the non‐injured side (VL: 381.23 ± 142.07 *vs* 603.9 ± 192.72, *P* < 0.001). In group C, the maximum voluntary contraction of both sides showed no differences in all muscles between bilateral limb (Table 3).

**Table 3:** The proprioceptive sense and maximum voluntary contraction in different group

	Group A			Group B			Group C		
	Injured side	Non‐injured side	*P*‐value	Injured side	Non‐injured side	*P*‐value	Injured side	Non‐injured side	*P*‐value
Tandem position	7.79 ± 1.57	6.33 ± 1.49	0.001*	6.64 ± 1.68	6.27 ± 1.34	0.551	6.08 ± 0.96	6.85 ± 3.47	0.302
Stance with one foot.	8.13 ± 0.84	7.1 ± 0.57	0.003*	7.03 ± 1.58	6.95 ± 1.72	0.895	7.33 ± 1.63	6.73 ± 1.92	0.388
Variance of 30°	6.96 ± 3.15	4.45 ± 1.67	0.030*	7.62 ± 4.98	4.33 ± 3.24	0.028*	8.61 ± 5.88	9.84 ± 6.68	0.469
Variance of 60°	4.64 ± 3.38	2.75 ± 1.98	0.044*	4.04 ± 2.19	3.09 ± 2.65	0.248	2.72 ± 2.07	2.47 ± 1.43	0.677
RF‐MVC	239.94 ± 129.70	364.81 ± 148.98	0.001*	459.48 ± 198.12	571.45 ± 273.38	0.075	254.67 ± 141.57	267.37 ± 125.78	0.715
VM‐MVC	298.88 ± 175.41	515.79 ± 272.49	0.001*	441.89 ± 175.04	521.25 ± 170.13	0.080	410.08 ± 226.97	342.77 ± 172.40	0.201
VL‐MVC	389.54 ± 157.97	594.28 ± 220.31	<0.001*	381.23 ± 142.07	603.9 ± 192.72	<0.001*	462.95 ± 148.36	396.48 ± 170.56	0.113

MVC, Maximum Voluntary Contraction; RF, rectus femoris; VL, vastus lateralis; VM, vastus medialis.

* There are significant differences.

In different training tasks, the RMS value was smaller for the injured side compared to the non‐injured side. In group A, the muscles of the injured side include RF, VM, and VL and are shown as obviously smaller in the single SLR (30°) training (RF: 190.1 ± 78.29 *vs* 256.73 ± 65.58, *P* = 0.001; VM: 173.61 ± 84.34 *vs* 362.65 ± 125.98, *P* = 0.001;VL: 487.58 ± 302.82 *vs* 259.13 ± 153.5, *P* = 0.003); only the VM and VL are shown as smaller in both SLR (30°) training (VM: 178.04 ± 81.09 *vs* 292.38 ± 148.93, *P* = 0.001; VL: 284.72 ± 81.73 *vs* 463.03 ± 99.64, *P* = 0.001); only the RF and VM are shown as smaller in the single SLR (60°) training (RF: 185.08 ± 44.27 *vs* 245.9 ± 30.93, *P* = 0.001; VM: 162.9 ± 62.56 *vs* 238.88 ± 75.07, *P* = 0.001); no muscle was shown as smaller in both SLR (60°) training, the VM and VL were shown as smaller in single ankle dorsiflexion training (VM: 122.28 ± 65.83 *vs* 249.53 ± 151.46, *P* = 0.001; VL: 163.38 ± 64.17 *vs* 369.68 ± 95.46, *P* = 0.001); the VL was shown to be smaller in both ankle dorsiflexion training (200.68 ± 115.61 *vs* 287.77 ± 122.16, *P* = 0.006). In group B, only the VL of the injury side was shown as obviously smaller in both‐SLR (30°) training (VL: 287.18 ± 106.76 *vs* 374.9 ± 103.22, *P* = 0.002). In group C, all of the muscles shown no significantly difference in all tasks (Table 4).

**Table 4:** The RMS value of the muscles in bilateral limb of difference training methods

	Single straight‐leg raising (30°)			Both straight‐leg raising (30°)			Single straight‐leg raising (60°)			Both straight‐leg raising (60°)			Single‐ankle dorsiflexion			Both‐ankle dorsiflexion		
	injured side	non‐injured side	*P*‐value	injured side	non‐injured side	*P*‐value	injured side	non‐injured side	*P*‐value	injured side	non‐injured side	*P*‐value	injured side	non‐injured side	*P*‐value	injured side	non‐injured side	*P*‐value
Group A																		
RF	190.1 ± 78.29	256.73 ± 65.58	0.001*	253.46 ± 192.84	227.18 ± 78.48	0.492	185.08 ± 44.27	245.9 ± 30.93	0.001*	159.1 ± 38.35	178.6 ± 46.85	0.083	125.33 ± 78.35	150.93 ± 91.17	0.248	130.93 ± 68.94	192.32 ± 267.59	0.229
VM	173.61 ± 84.34	362.65 ± 125.98	0.001*	178.04 ± 81.09	292.38 ± 148.93	0.001*	162.9 ± 62.56	238.88 ± 75.07	0.001*	132.78 ± 65.14	168.73 ± 97.44	0.098	122.28 ± 65.83	249.53 ± 151.46	0.001*	198.09 ± 79.71	231.68 ± 81.56	0.112
VL	259.13 ± 153.5	487.58 ± 302.82	0.003*	284.72 ± 81.73	463.03 ± 99.64	0.001*	199.23 ± 59.69	230.5 ± 78.19	0.087	148.88 ± 50.79	172.68 ± 75.08	0.156	163.38 ± 64.17	369.68 ± 95.46	0.001*	200.68 ± 115.61	287.77 ± 122.16	0.006*
Group B																		
RF	269.35 ± 144.44	350.22 ± 198.12	0.076	255.18 ± 104.64	281.9 ± 121.66	0.365	169.63 ± 54.45	170.8 ± 43.14	0.927	124.13 ± 21.79	126.8 ± 40.15	0.750	164.38 ± 45.66	155.1 ± 52.19	0.467	201.33 ± 79.01	176.02 ± 50.40	0.145
VM	309.0 ± 120.07	360.33 ± 159.96	0.153	274.78 ± 103.48	321.3 ± 128.87	0.129	213.8 ± 66.03	241.03 ± 59.68	0.099	179.13 ± 84.94	203.83 ± 77.81	0.245	233.52 ± 62.75	205.55 ± 86.79	0.158	216.15 ± 61.52	196.07 ± 44.06	0.152
VL	382.7 ± 136.09	439.62 ± 119.74	0.091	287.18 ± 106.76	374.9 ± 103.22	0.002*	363.7 ± 98.95	408.1 ± 83.12	0.065	243.77 ± 91.62	281.47 ± 84.63	0.103	258.88 ± 79.23	231.9 ± 91.51	0.227	248.63 ± 86.51	217.35 ± 79.63	0.151
Group C																		
RF	269.16 ± 74.43	310.44 ± 93.12	0.063	213.53 ± 92.05	267.27 ± 139.1	0.083	175.21 ± 75.98	174.12 ± 72.31	0.955	141.34 ± 42.76	151.76 ± 48.53	0.381	148.78 ± 64.18	176.27 ± 72.99	0.127	130.12 ± 52.13	126.58 ± 48.24	0.786
VM	317.4 ± 107.53	363.18 ± 93.13	0.083	410.05 ± 84.71	415.88 ± 79.15	0.784	209.88 ± 70.87	241.46 ± 76.42	0.102	197.53 ± 67.29	201.58 ± 51.12	0.794	179.88 ± 93.63	226.92 ± 108.85	0.078	186.38 ± 85.95	187.42 ± 57.74	0.956
VL	387.14 ± 103.29	426.58 ± 91.24	0.122	386.25 ± 105.94	432.68 ± 107.13	0.097	398.64 ± 72.98	412.72 ± 64.37	0.395	287.67 ± 79.43	311.54 ± 68.74	0.218	168.55 ± 81.19	208.57 ± 106.58	0.107	209.08 ± 98.48	251.38 ± 102.11	0.108

MVC, Maximum Voluntary Contraction; RF, rectus femoris; VL, vastus lateralis; VM, vastus medialis.

* There are significant differences.

### *The Gait Measurement Results*

In the test for walking ability, eight walking models and 15 parameters in each model were used for analysis. The divergences between bilateral limb were found in different groups. In group A, there were 26 parameters that had significant differences between both sides. The 26 parameters include five in walking, four in fast walking, four in serpentine walk, three in upstairs, two in inverted walking, two in downstairs, one in walking with double task, and one in normal walking after warm‐up (Table S1). In group B, there were eight parameters that showed significant differences between both sides. The eight parameters include two in walking, two in downstairs, one in serpentine walk, one in upstairs, one in inverted walking, and one in normal walking after warm‐up (Table S2). In group C, there were 16 parameters that differentiate including six in walking, three in downstairs, three in normal walking after warm‐up, two in walking with double task, one in fast walking, and one in serpentine walking (Table S3).

### *The Proprioception Measurement Results*

**Figure 2:**
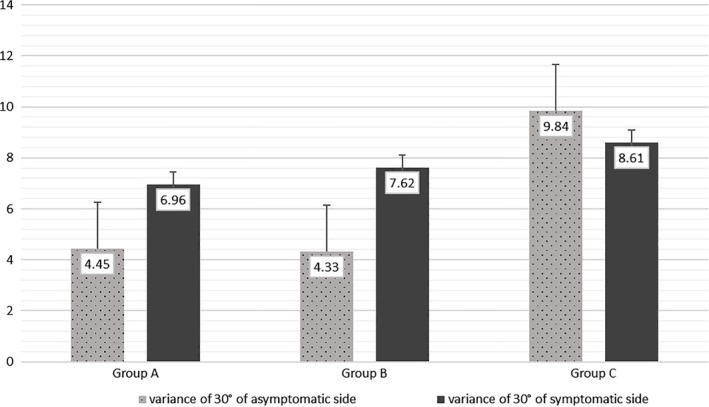
The variance of proprioceptive sense in 30° of different group. The variance of proprioception in 30° of noninjured side became larger gradually, and the variance of proprioception in 30° of injured side shown no significantly changes.

In the proprioception test, the division between the actual test results and the set values of injured side was significantly larger when compared to the non‐injury side. In group A, all of the proprioception measurement of injury sides was larger when compared to the non‐injury sides (tandem position: 7.79 ± 1.57 *vs* 6.33 ± 1.49, *P* = 0.001; stance with one foot: 8.13 ± 0.84 *vs* 7.1 ± 0.57, *P* = 0.003; variance of 30°: 6.96 ± 3.15 *vs* 4.45 ± 1.67, *P* = 0.03; variance of 60°: 4.64 ± 3.38 *vs* 2.75 ± 1.98, *P* = 0.044). In group B, only variance of 30° of injury side showed as being larger when compared to the non‐injury side (7.62 ± 4.98 *vs* 4.33 ± 3.24, *P* = 0.028). In group C, there are no differences in parameters between bilateral sides (Table 3); however, the location assessment (30°) results of group C showed no statistical differences, which is not because the affected side got “better”, but because the healthy side get “worse”. The proprioception in 30° of non‐injured side in group C was worse than group B (9.84 ± 6.68 *vs* 4.33 ± 3.24, *P* < 0.001) and group A (9.84 ± 6.68 *vs* 4.45 ± 1.67, *P* < 0.001), and there were no differences between each group in the injured side (Table 3, Fig. 2).

## Discussion

The purpose of this study was to show the biomechanics characteristics of patients with ACL injury, and then provide a rehabilitation suggestion based on the results. We found that the proprioception at 30° of the injured side will not recover and the non‐injury side will become worse after 1 year from the injury. We also found the VL, VM, and RF had different activated rules in different groups, and the recovery of VL was not satisfied as VM and RF. In the data of gait analysis, we found that the gait stability will become worse after 1 year from injury. In the gait analysis, the best periods of gait stability were 1.5 months to 1 year. After 1 year, the patient's gait stability will gradually decline again. These findings suggest that the training for proprioception at 30° and VL are important for rehabilitation, and without surgical treatment, the knee stability will become worse after 1 year.

### *Characteristics of Muscles and Their Effect on Rehabilitation*

The pre‐operative rehabilitation is extremely important to patients' functional outcome^21^, ^22^. Current studies confirmed that the muscle atrophy will start soon after the injury of ACL, and the main purpose of rehabilitation is to prevent muscle atrophy, especially quadriceps femoris atrophy^23^, ^24^. Although many researchers proved that training the muscle is important for the rehabilitation, the best training methods have not been clearly clarified^25^, ^26^, ^27^. In this study, we found that in early stage (3 weeks–1.5 months) after injury, all of the muscles include RF, VM, and VL decreased significantly and the single SLR (30°) may be the best method to training muscle function. In the middle stage (1.5 months–1 year) after injury, all the muscles recovered to the same level as non‐involved side, except the VL; therefore, the special training for the VL would be recommended to improve the overall function at this stage. Meanwhile, both SLR (30°) can only improve the VL when compared to other methods suggested in this study. In the later stage (after 1 year), all of the muscles were recovered at the same level as the non‐injured side and all the tasks in this study cannot train the muscle function (Table 2).

The SLR is a widely used training method on the bed, and different lift angles or rotation angles can activate different muscles. A study by Babadi *et al*.^28^ indicated performing SLR exercise with hip external rotation was the most effective method for vastus medialis oblique. A study by Mikaili *et al*.^29^ demonstrated that SLR training with hip external hip rotation and the contraction of ankle dorsiflexors is the best way to train the quadriceps muscle. Santos's^30^ study showed that knee extensors and flexors can increase the muscle thickness of both VM and VL. In general, the single SLR can be accomplished by activating the RF^31^. However, the bilateral SLR training needs twice the force when compared to single SLR training (principle of linear dependence) and more muscles needed to be activated so as to accomplish the task^32^. Therefore, the VL may be better activated only in this high‐intensity training.

### *Characteristics of Proprioception and Their Effect on Rehabilitation*

The injury of ACL can also seriously affect the proprioception of the lower limb^33^. In this study, we proved that all the proprioception decreased seriously just after injury and the majority of patients can recover within 1 year except when the location perception is at 30°. After 1 year, the gap between the actual results and the set values of location perception of 30° showed continuous increases in the non‐injury side. This is the new finding in this study, as the location perception at 30° in the injured side wasn't getting “better” but the non‐injury side got “worse” as time went on. The statistical results showed that the location perception at 30° of the injured side and the non‐injured side got to a similar level after 1 year of the injury occurring (Table 1). The damage of location perception at 30° may be caused by the function disorder of ACL in the injured side. At a lower angle, the femur tends to be backward relative to the tibia. During this angle range, the stability of the knee joint is maintained mainly by ACL. Therefore, the tibia has internal rotation and internal displacement relative to the femur at a lower angle. As the angle increases gradually and exceeds 30°, the tibial rotation and forward movement gradually decreased or disappeared and the femur tended to be forward relative to tibia. At this time, the stability of the knee joint was mainly maintained by posterior cruciate ligament (PCL)^34^, ^35^.

The reason lead to the non‐injured side turning “bad”, which was potentially caused by the cross‐transport effect. This phenomenon had already been found in upper limb training^36^ and patients of stroke^37^. A study by Farthing *et al*.^36^ also determined the contralateral arm training can counteract muscle atrophy caused by limb fixation. Meanwhile, Grooms *et al*.^38^ thought it may be due to the alternation of brain function, but the effect of changes in brain function on lower limb balance is not clear. Anyway, the special training for the location perception at 30° before operation is very important for the functional rehabilitation.

### *Characteristics of Gait and Their Effect on Rehabilitation*

Walking is a human behavior that requires coordination of movement and equilibrium of muscles and joints. Any injury of muscle and joints will lead to gait disorder^39^. The injury of ACL will not only affect the gait stability of patients, but also changes the biomechanical environment of the knee joint^40^. Osteoarthritis and meniscus injury are two types of complications that are commonly found in ACL injury patients^41^. In this study, we also found that the gait stability was decreasing again after 1 year from ACL injury. This phenomenon may suggest that the complications caused by ACL injury will appear and influence walking ability after 1 year from injury. Therefore, it is suggested to conduct the ACL reconstruction surgery within 1 year from the injury.

### *Limitations*

The present study has been conducted on only 90 patients, and this limited sample reduces the informative value of the study. The imprecise grouping of samples also reduces the representativeness of results. Therefore, we will continue to expand the sample of the study, improve the grouping ability, and provided more evidence to verify the results.

### *Conclusion*

This study examined the gait, SEMG, and proprioception characteristics of the patients with ACL injury for different stages. The results proved the deterioration of proprioception in 30° of injured side will not be recovered, and the non‐injury side will become worse after 1 year from injury. Among the VL, VM, and RF, the recovery rate of VL is the slowest and the bilateral SLR (30°) is the best way to train it. Specifically, we found that the gait stability will become worse again after 1 year from the injury. Therefore, we suggest that the training for proprioception in 30° and VL are important for the rehabilitation, and the ACL reconstruction should be performed within 1 year of injury.

## Supporting information


**Table S1** The Results of Gait Analysis in Group A.

**Table S2** The Results of Gait Analysis in Group B.

**Table S3** The Results of Gait Analysis in Group C.
