# Supplementary material for: Biomechanical Evaluation of Preoperative Rehabilitation in Patients of Anterior Cruciate Ligament Injury
Source: Orthop Surg. 2020 Mar 8;12(2):421–8. doi: 10.1111/os.12607 (PMC7189052; doi:10.1111/os.12607)
Supplement: Supplementary file 1 — Table S1 The Results of Gait Analysis in Group A. [file OS-12-421-s001.docx]

| **Supplement Table 1** The Results of Gait Analysis in Group A | | | | | | | | | | | | | | | | | | | | | | | | |
| --- | --- | --- | --- | --- | --- | --- | --- | --- | --- | --- | --- | --- | --- | --- | --- | --- | --- | --- | --- | --- | --- | --- | --- | --- |
|  | walking | | | Fast walking | | | Inverted walking | | | Serpentine walk | | | walking with double task | | | Upstairs | | | Downstairs | | | normal walking after warm-up | | |
|  | injured side | non-injured side | P | injured side | non-injured side | P | injured side | non-injured side | P | injured side | non-injured side | P | injured side | non-injured side | P | injured side | non-injured side | P | injured side | non-injured side | P | injured side | non-injured side | P |
| Single Support (ms) | 434.59±28.54 | 441.43±39.66 | 0.446 | 359.63±32.60 | 367.65±35.40 | 0.365 | 220.26±127.09 | 279.06±170.09 | 0.135 | 426.67±76.39 | 439.35±66.33 | 0.495 | 415.3±43.05 | 413.7±33.01 | 0.872 | 506.04±174.28 | 571.38±189.69 | 0.172 | 488.54±97.05 | 488.67±85.75 | 0.996 | 423.93±41.27 | 446.45±48.88 | 0.059 |
| Double Support (ms) | 137.11±16.24 | 146.62±19.63 | 0.045* | 118.88±22.66 | 123.8±26.91 | 0.447 | 137.4±71.96 | 135.4±50.42 | 0.901 | 126.05±24.28 | 132.78±28.48 | 0.329 | 127±18.05 | 138.51±26.47 | 0.054 | 118.96±46.52 | 115.89±29.26 | 0.761 | 141.86±24.12 | 128.19±29.36 | 0.054 | 129.79±22.10 | 138.07±13.79 | 0.087 |
| SLS/DLS (%) | 95.23±24.21 | 109.17±25.66 | 0.035* | 93.15±29.38 | 109.03±30.87 | 0.046* | 151.01±31.82 | 134.37±41.96 | 0.089 | 130.56±34.81 | 151.22±41.75 | 0.052 | 128.08±41.69 | 133.15±37.63 | 0.623 | 172.23±98.98 | 212.71±148.49 | 0.219 | 187.33±59.22 | 233.43±68.59 | 0.007* | 142.81±36.92 | 151.33±35.54 | 0.366 |
| Swing Duration (ms) | 440.32±28.19 | 411.84±24.03 | 0.001* | 394.74±25.47 | 374.97±21.41 | 0.002* | 746.31±117.37 | 701.6±102.96 | 0.122 | 444.69±41.91 | 411.27±35.39 | 0.002* | 421.69±18.55 | 402.87±14.71 | <0.001* | 501.66±130.59 | 386.38±51.86 | <0.001* | 542.07±146.16 | 562.19±221.59 | 0.679 | 439.24±20.04 | 411.51±15.75 | <0.001* |
| Step Duration (ms) | 577.31±53.18 | 562.6±39.01 | 0.227 | 499.21±53.96 | 470.95±50.87 | 0.041* | 1022.1±289.92 | 903.44±215.69 | 0.077 | 571.2±85.74 | 550.48±100.18 | 0.393 | 540.81±52.75 | 545.53±58.81 | 0.744 | 751.31±240.00 | 665.85±205.89 | 0.144 | 661.01±117.79 | 628.53±101.99 | 0.258 | 575.19±56.13 | 556.6±42.02 | 0.152 |
| Cycle Duration (sec) | 1.16±0.09 | 1.15±0.09 | 0.668 | 0.97±0.11 | 0.96±0.11 | 0.726 | 1.79±0.43 | 1.75±0.44 | 0.723 | 1.13±0.18 | 1.14±0.19 | 0.835 | 1.08±0.10 | 1.09±0.11 | 0.714 | 1.45±0.43 | 1.35±0.36 | 0.333 | 1.39±0.46 | 1.42±0.44 | 0.797 | 1.14±0.09 | 1.15±0.10 | 0.685 |
| Pulling Accel. (G) | 1.26±0.38 | 1.43±0.25 | 0.044* | 1.82±0.66 | 1.95±0.61 | 0.431 | 1.92±0.85 | 1.87±0.78 | 0.813 | 1.34±0.54 | 1.47±0.53 | 0.351 | 1.45±0.38 | 1.49±0.26 | 0.636 | 0.83±0.41 | 0.82±0.42 | 0.926 | 0.86±0.35 | 0.92±0.31 | 0.485 | 1.25±0.40 | 1.24±0.37 | 0.92 |
| Swing Power (G) | 0.54±0.18 | 0.67±0.19 | 0.086 | 0.87±0.28 | 0.94±0.35 | 0.396 | 0.70±0.25 | 0.83±0.28 | 0.063 | 0.51±0.18 | 0.63±0.23 | 0.028* | 0.67±0.16 | 0.73±0.19 | 0.191 | 0.61±0.36 | 0.80±0.42 | 0.065 | 0.45±0.29 | 0.56±0.30 | 0.154 | 0.58±0.15 | 0.65±0.12 | 0.051 |
| Ground Impact (G) | 1.32±0.39 | 1.51±0.25 | 0.028 | 1.84±0.37 | 1.98±0.29 | 0.108 | 0.45±0.18 | 0.64±0.28 | 0.003* | 1.24±0.29 | 1.45±0.27 | 0.005* | 1.52±0.31 | 1.65±0.21 | 0.062 | 0.81±0.31 | 1.33±0.74 | 0.001* | 0.92±0.44 | 1.01±0.31 | 0.364 | 1.31±0.30 | 1.46±0.29 | 0.054 |
| Foot fall | 3.09±0.82 | 3.42±0.51 | 0.066 | 4.48±1.04 | 4.71±0.73 | 0.326 | 1.61±0.68 | 1.64±0.43 | 0.839 | 2.97±0.81 | 3.37±0.78 | 0.056 | 3.58±0.69 | 3.83±0.44 | 0.099 | 1.76±0.70 | 2.80±1.31 | 0.001* | 2.01±1.02 | 2.25±0.69 | 0.29 | 3.06±0.71 | 3.34±0.35 | 0.058 |
| Push off | 17.15±5.26 | 18.77±7.16 | 0.321 | 25.82±8.21 | 27.38±11.63 | 0.551 | 51.98±21.99 | 40.41±25.84 | 0.065 | 18.06±6.44 | 22.09±10.53 | 0.079 | 21.96±8.28 | 20.39±6.56 | 0.419 | 13.74±5.12 | 16.41±6.57 | 0.084 | 10.48±5.51 | 15.89±8.58 | 0.005* | 16.89±9.12 | 21.73±10.54 | 0.062 |
| Speed (m/min) | 60.81±9.58 | 62.95±9.20 | 0.381 | 91.91±11.66 | 89.72±18.16 | 0.581 | 40.73±15.79 | 48.92±21.08 | 0.094 | 63.41±16.14 | 66.95±19.66 | 0.449 | 71.33±13.79 | 70.78±14.11 | 0.879 | 40.66±11.99 | 46.12±16.34 | 0.146 | 44.91±14.76 | 45.53±10.78 | 0.853 | 61.6±12.95 | 63.2±11.82 | 0.619 |
| Cadence (steps/min) | 103.18±8.51 | 105.76±6.57 | 0.194 | 121.43±14.81 | 129.87±14.02 | 0.027* | 70.26±20.21 | 86.73±24.49 | 0.006* | 106.99±15.89 | 119.38±19.05 | 0.008* | 110.11±9.89 | 111.66±8.64 | 0.521 | 86.78±19.69 | 94.86±20.47 | 0.125 | 99.73±14.59 | 99.3±10.00 | 0.895 | 104.12±8.99 | 107.25±7.14 | 0.141 |
| Step Length (meters) | 0.59±0.05 | 0.60±0.06 | 0.486 | 0.72±0.08 | 0.73±0.09 | 0.651 | 0.68±0.04 | 0.66±0.05 | 0.093 | 0.59±0.08 | 0.58±0.08 | 0.63 | 0.64±0.08 | 0.65±0.08 | 0.63 | 0.48±0.09 | 0.49±0.10 | 0.685 | 0.45±0.08 | 0.47±0.08 | 0.337 | 0.59±0.08 | 0.58±0.08 | 0.63 |
| Stride Length (meters) | 1.18±0.11 | 1.19±0.11 | 0.726 | 1.45±0.17 | 1.46±0.19 | 0.831 | 1.15±0.21 | 1.04±0.28 | 0.091 | 1.19±0.16 | 1.18±0.16 | 0.81 | 1.29±0.16 | 1.28±0.15 | 0.804 | 0.89±0.11 | 0.86±0.16 | 0.401 | 0.87±0.20 | 0.83±0.21 | 0.453 | 1.18±0.16 | 1.19±0.16 | 0.81 |
