# Supplementary material for: Biomechanical Evaluation of Preoperative Rehabilitation in Patients of Anterior Cruciate Ligament Injury
Source: Orthop Surg. 2020 Mar 8;12(2):421–8. doi: 10.1111/os.12607 (PMC7189052; doi:10.1111/os.12607)
Supplement: Supplementary file 2 — Table S2 The Results of Gait Analysis in Group B. [file OS-12-421-s002.docx]

| **Table 4**  The Results of Gait Analysis in Group B | | | | | | | | | | | | | | | | | | | | | | | | |
| --- | --- | --- | --- | --- | --- | --- | --- | --- | --- | --- | --- | --- | --- | --- | --- | --- | --- | --- | --- | --- | --- | --- | --- | --- |
|  | walking | | | Fast walking | | | Inverted walking | | | Serpentine walk | | | walking with double task | | | Upstairs | | | Downstairs | | | normal walking after warm-up | | |
|  | injured side | non-injured side | P | injured side | non-injured side | P | injured side | non-injured side | P | injured side | non-injured side | P | injured side | non-injured side | P | injured side | non-injured side | P | injured side | non-injured side | P | injured side | non-injured side | P |
| Single Support (ms) | 416.19±25.49 | 417.73±20.95 | 0.799 | 362.53±33.35 | 363.71±30.41 | 0.887 | 271.26±149.39 | 293.37±160.62 | 0.583 | 415.15±43.56 | 418.2±43.34 | 0.787 | 405.53±28.50 | 410.7±36.39 | 0.543 | 542.73±97.22 | 499.11±77.93 | 0.074 | 458.34±81.42 | 426.31±50.76 | 0.073 | 427.96±37.67 | 424.39±40.75 | 0.726 |
| Double Support (ms) | 142.25±11.20 | 141.44±12.11 | 0.789 | 122.83±15.04 | 125.86±15.71 | 0.448 | 134.69±34.58 | 154.37±45.12 | 0.063 | 131.65±15.75 | 138.14±12.61 | 0.083 | 137.03±13.03 | 134.43±15.94 | 0.492 | 113.78±27.86 | 125.78±25.81 | 0.089 | 123.69±36.08 | 109.12±29.79 | 0.093 | 135.64±5.04 | 139.63±16.75 | 0.217 |
| SLS/DLS (%) | 141.51±54.98 | 159.82±59.91 | 0.214 | 102.42±24.03 | 102.64±28.51 | 0.974 | 146.99±32.01 | 161.56±37.49 | 0.111 | 129.81±30.82 | 134.97±36.56 | 0.557 | 116.83±27.88 | 125.1±28 | 0.256 | 195.3±68.31 | 142.33±43.72 | 0.001* | 139.58±52.88 | 164.58±63.77 | 0.104 | 135.18±32.82 | 144.24±30.73 | 0.274 |
| Swing Duration (ms) | 428.34±18.79 | 418.13±19.61 | 0.044* | 390.49±26.71 | 384.96±25.24 | 0.413 | 703.57±161.04 | 707.13±177.01 | 0.935 | 426.6±27.33 | 412.48±31.13 | 0.067 | 426.81±22.21 | 417.13±19.71 | 0.079 | 432.44±59.46 | 440.21±58.34 | 0.611 | 490.83±59.74 | 461.84±64.69 | 0.077 | 429.13±28.86 | 416.38±24.23 | 0.069 |
| Step Duration (ms) | 551.32±28.43 | 549.84±32.32 | 0.851 | 486.94±37.44 | 491.48±47.46 | 0.683 | 741.33±156.07 | 774.03±211.79 | 0.499 | 549.39±51.62 | 540.64±49.89 | 0.507 | 535.94±45.84 | 536.07±42.43 | 0.991 | 636.07±112.38 | 617.51±91.06 | 0.485 | 657.57±59.37 | 624.88±68.25 | 0.053 | 561.82±54.15 | 553.08±38.29 | 0.473 |
| Cycle Duration (sec) | 1.12±0.21 | 1.11±0.22 | 0.077 | 0.98±0.08 | 0.99±0.08 | 0.63 | 1.39±0.28 | 1.50±0.51 | 0.305 | 1.08±0.1 | 1.09±0.09 | 0.685 | 1.08±0.09 | 1.09±0.09 | 0.669 | 1.29±0.17 | 1.38±0.45 | 0.31 | 1.27±0.60 | 1.11±0.19 | 0.169 | 1.12±0.08 | 1.11±0.08 | 0.63 |
| Pulling Accel. (G) | 1.01±0.37 | 1.15±0.28 | 0.104 | 1.39±0.59 | 1.50±0.53 | 0.451 | 1.77±1.16 | 1.56±1.01 | 0.458 | 1.02±.038 | 1.14±0.32 | 0.191 | 1.18±0.66 | 1.19±0.32 | 0.941 | 0.86±.024 | 0.82±0.26 | 0.538 | 1.16±0.48 | 1.10±0.42 | 0.608 | 1.09±0.50 | 1.04±0.34 | 0.652 |
| Swing Power (G) | 0.62±0.19 | 0.71±0.17 | 0.058 | 0.88±0.24 | 0.93±0.31 | 0.488 | 0.67±0.25 | 0.76±0.31 | 0.221 | 0.59±0.12 | 0.65±0.13 | 0.068 | 0.69±0.24 | 0.81±0.29 | 0.086 | 0.84±0.39 | 0.75±0.22 | 0.276 | 0.56±0.16 | 0.65±0.19 | 0.052 | 0.59±0.18 | 0.66±0.12 | 0.081 |
| Ground Impact (G) | 1.44±0.43 | 1.62±0.41 | 0.102 | 1.81±0.44 | 1.71±0.45 | 0.388 | 0.71±0.29 | 0.88±0.32 | 0.035* | 1.29±0.21 | 1.48±0.20 | 0.001* | 1.51±0.31 | 1.66±0.35 | 0.084 | 1.39±0.30 | 1.5±0.16 | 0.082 | 0.98±0.37 | 1.21±0.57 | 0.069 | 1.33±0.34 | 1.47±0.19 | 0.054 |
| Foot fall | 3.40±0.81 | 3.76±0.76 | 0.081 | 4.49±0.87 | 4.65±0.81 | 0.464 | 1.85±0.91 | 2.24±1.11 | 0.142 | 3.25±0.43 | 3.46±0.54 | 0.101 | 3.61±0.82 | 4.01±0.81 | 0.062 | 2.77±0.79 | 3.13±0.97 | 0.12 | 2.24±1.02 | 2.79±1.12 | 0.052 | 3.28±0.68 | 3.52±0.42 | 0.105 |
| Push off | 23.42±12.56 | 24.38±12.7 | 0.769 | 32.25±12.68 | 35.18±17.65 | 0.463 | 45.14±18.18 | 41.64±21.43 | 0.407 | 23.36±13.02 | 25.99±16.31 | 0.493 | 21.47±9.99 | 21.53±9.13 | 0.981 | 16.63±8.07 | 16.04±7.04 | 0.764 | 9.86±6.54 | 17.32±7.93 | 0.001* | 20.51±10.67 | 24.51±11.44 | 0.167 |
| Speed (m/min) | 71.31±7.19 | 70.2±7.31 | 0.556 | 95.94±14.54 | 93.34±16.34 | 0.518 | 56.06±13.61 | 56.81±20.15 | 0.866 | 69.57±10.18 | 70.54±10.11 | 0.713 | 75.91±8.46 | 74.04±6.21 | 0.333 | 49.17±9.35 | 55.76±16.80 | 0.066 | 61.5±20.86 | 53.14±18.05 | 0.102 | 65.63±5.51 | 65.42±8.28 | 0.908 |
| Cadence (steps/min) | 107.33±4.91 | 107.85±6.21 | 0.72 | 123.41±9.33 | 124.07±10.46 | 0.797 | 92±23.11 | 93.83±21.77 | 0.753 | 108.81±10.63 | 110.94±11.22 | 0.453 | 110.47±9.41 | 110.67±8.65 | 0.932 | 95.14±13.20 | 97.79±14.59 | 0.464 | 114.17±8.20 | 110.74±13.33 | 0.235 | 107.06±9.77 | 108.21±7.55 | 0.612 |
| Step Length (meters) | 0.68±0.06 | 0.65±0.05 | 0.04* | 0.78±0.08 | 0.77±0.08 | 0.63 | 0.66±0.05 | 0.64±0.04 | 0.093 | 0.64±0.05 | 0.63±0.05 | 0.442 | 0.69±0.06 | 0.67±0.04 | 0.134 | 0.52±0.06 | 0.55±0.09 | 0.134 | 0.55±0.05 | 0.47±0.05 | <0.001* | 0.64±0.05 | 0.61±0.06 | 0.04* |
| Stride Length (meters) | 1.31±0.11 | 1.32±0.11 | 0.726 | 1.54±0.16 | 1.55±0.17 | 0.815 | 1.11±0.17 | 1.15±0.19 | 0.394 | 1.28±0.09 | 1.27±0.09 | 0.669 | 1.36±0.09 | 1.35±0.08 | 0.651 | 1.01±0.06 | 0.94±0.30 | 0.215 | 1.05±0.10 | 0.93±0.14 | 0.001* | 1.23±0.12 | 1.24±0.11 | 0.738 |
