# Supplementary material for: Biomechanical Evaluation of Preoperative Rehabilitation in Patients of Anterior Cruciate Ligament Injury
Source: Orthop Surg. 2020 Mar 8;12(2):421–8. doi: 10.1111/os.12607 (PMC7189052; doi:10.1111/os.12607)
Supplement: Supplementary file 3 — Table S3 The Results of Gait Analysis in Group C. [file OS-12-421-s003.docx]

| **Table 5**  The Results of Gait Analysis in Group C | | | | | | | | | | | | | | | | | | | | | | | | |
| --- | --- | --- | --- | --- | --- | --- | --- | --- | --- | --- | --- | --- | --- | --- | --- | --- | --- | --- | --- | --- | --- | --- | --- | --- |
|  | walking | | | Fast walking | | | Inverted walking | | | Serpentine walk | | | walking with double task | | | Upstairs | | | Downstairs | | | normal walking after warm-up | | |
|  | injured side | non-injured side | P | injured side | non-injured side | P | injured side | non-injured side | P | injured side | non-injured side | P | injured side | non-injured side | P | injured side | non-injured side | P | injured side | non-injured side | P | injured side | non-injured side | P |
| Single Support (ms) | 406.88±39.34 | 429.13±42.51 | 0.04* | 352.65±31.69 | 356.68±28.73 | 0.608 | 143.45±75.53 | 172.2±95.87 | 0.202 | 390.19±31.88 | 397.75±30.18 | 0.349 | 385.06±13.32 | 387.38±14.88 | 0.527 | 510±132.52 | 493.07±139.06 | 0.631 | 458.34±81.42 | 426.31±50.76 | 0.073 | 391.42±84.37 | 422.81±35.12 | 0.065 |
| Double Support (ms) | 140.1±15.55 | 138.63±8.79 | 0.654 | 118.79±9.68 | 121.46±9.69 | 0.29 | 134.73±78.31 | 128.8±59.32 | 0.742 | 123.35±18.59 | 123.94±15.52 | 0.894 | 138.68±15.85 | 132.48±12.05 | 0.093 | 122.4±30.96 | 136.72±26.63 | 0.06 | 122.59±36.08 | 109.12±26.79 | 0.106 | 131.65±18.94 | 137.53±16.64 | 0.207 |
| SLS/DLS (%) | 131.65±52.43 | 172.46±71.46 | 0.512 | 94.53±12.14 | 96.94±18.85 | 0.558 | 143.7±64.51 | 175.03±69.78 | 0.076 | 114.2±19.18 | 117.54±19.95 | 0.511 | 111.36±10.03 | 125.24±11.41 | <0.001* | 157.54±38.48 | 167.98±66.10 | 0.458 | 139.58±42.88 | 164.58±63.77 | 0.08 | 137.43±23.19 | 144.14±21.38 | 0.249 |
| Swing Duration (ms) | 431.62±32.07 | 413.08±23.50 | 0.013* | 385.3±24.45 | 372.04±22.96 | 0.035* | 806.35±89.83 | 768.65±91.33 | 0.112 | 409.81±22.44 | 398.39±21.32 | 0.048* | 405.96±14.25 | 399.2±13.37 | 0.063 | 481.34±49.6 | 462.27±33.24 | 0.086 | 498.83±79.74 | 459.84±84.69 | 0.072 | 426.18±34.29 | 408.04±24.09 | 0.022* |
| Step Duration (ms) | 564.79±45.35 | 540.48±37.61 | 0.028* | 478.01±21.75 | 474.4±37.47 | 0.65 | 810.08±32.76 | 808.43±40.49 | 0.863 | 513.64±33.22 | 510.3±46.50 | 0.75 | 508.9±15.11 | 517.3±14.79 | 0.034* | 672.21±88.85 | 631.52±68.33 | 0.052 | 627.57±89.37 | 599.88±78.25 | 0.207 | 551.28±50.09 | 542.84±36.13 | 0.457 |
| Cycle Duration (sec) | 1.13±0.09 | 1.11±0.08 | 0.367 | 0.95±0.05 | 0.96±0.06 | 0.486 | 1.51±0.32 | 1.62±0.31 | 0.182 | 1.02±0.07 | 1.03±0.08 | 0.608 | 1.04±0.04 | 1.03±0.03 | 0.278 | 1.39±0.37 | 1.25±0.18 | 0.067 | 1.27±0.60 | 1.11±0.19 | 0.169 | 1.10±0.08 | 1.11±0.08 | 0.63 |
| Pulling Accel. (G) | 1.12±0.29 | 1.06±0.31 | 0.442 | 1.59±0.41 | 1.63±0.44 | 0.717 | 2.21±0.44 | 1.98±0.46 | 0.053 | 1.37±0.34 | 1.33±0.46 | 0.703 | 1.38±0.42 | 1.39±0.62 | 0.942 | 0.94±0.43 | 0.75±0.46 | 0.104 | 1.16±0.48 | 1.10±0.42 | 0.608 | 1.11±0.28 | 1.22±0.47 | 0.275 |
| Swing Power (G) | 0.61±0.27 | 0.65±0.21 | 0.524 | 0.94±0.29 | 1.02±0.32 | 0.315 | 0.97±0.23 | 1.04±0.24 | 0.254 | 0.73±0.26 | 0.75±0.23 | 0.754 | 0.82±0.21 | 0.85±0.16 | 0.536 | 0.85±0.39 | 0.74±0.24 | 0.194 | 0.59±0.16 | 0.71±0.39 | 0.124 | 0.64±0.24 | 0.73±0.16 | 0.093 |
| Ground Impact (G) | 1.47±0.44 | 1.55±0.33 | 0.429 | 2.13±0.59 | 2.32±0.61 | 0.225 | 0.46±0.08 | 0.51±0.12 | 0.063 | 1.58±0.40 | 1.71±0.35 | 0.186 | 1.77±0.31 | 1.94±0.35 | 0.051 | 1.24±0.57 | 1.35±0.52 | 0.438 | 0.98±0.47 | 1.23±0.57 | 0.069 | 1.43±0.4 | 1.57±0.25 | 0.109 |
| Foot fall | 3.51±0.97 | 3.57±0.64 | 0.778 | 4.82±0.87 | 4.98±0.95 | 0.499 | 1.12±0.60 | 0.96±0.31 | 0.199 | 3.89±0.88 | 3.92±0.75 | 0.887 | 4.09±0.54 | 4.19±0.58 | 0.492 | 2.62±1.17 | 2.83±1.11 | 0.479 | 2.28±0.97 | 2.79±1.12 | 0.064 | 3.47±0.87 | 3.71±0.53 | 0.202 |
| Push off | 24.22±9.98 | 32.18±16.29 | 0.026* | 38.88±18.81 | 38.85±14.27 | 0.995 | 54.35±18.89 | 60.58±18.48 | 0.202 | 26.17±11.62 | 32.24±14.19 | 0.075 | 19.56±1.97 | 22.84±9.30 | 0.064 | 19.57±3.99 | 20.87±3.04 | 0.161 | 9.86±6.54 | 17.32±7.94 | 0.001* | 26.03±9.84 | 30.61±10.91 | 0.093 |
| Speed (m/min) | 72.04±11.05 | 71.26±10.44 | 0.779 | 101.91±14.09 | 101.06±16.03 | 0.828 | 50.25±19.23 | 54.43±21.03 | 0.425 | 81.62±12.15 | 81.28±13.63 | 0.919 | 81.2±7.13 | 80.4±8.06 | 0.685 | 50.38±11.42 | 48.50±7.54 | 0.455 | 61.5±20.86 | 53.14±18.05 | 0.102 | 71.39±8.84 | 70.13±7.41 | 0.552 |
| Cadence (steps/min) | 107.19±7.36 | 108.95±6.94 | 0.334 | 125.12±6.65 | 127.87±10.05 | 0.216 | 88.18±33.18 | 82.45±16.32 | 0.399 | 115.65±7.51 | 117.11±9.16 | 0.502 | 115.86±3.81 | 114.14±3.51 | 0.074 | 94.98±16.47 | 96.56±10.23 | 0.87 | 114.17±8.20 | 110.74±13.33 | 0.235 | 107.74±7.97 | 111.97±6.74 | 0.03* |
| Step Length (meters) | 0.68±0.08 | 0.63±0.08 | 0.019* | 0.81±0.09 | 0.82±0.09 | 0.669 | 0.62±0.03 | 0.65±0.09 | 0.089 | 0.71±0.09 | 0.69±0.08 | 0.367 | 0.70±0.06 | 0.71±0.06 | 0.521 | 0.55±0.08 | 0.52±0.07 | 0.128 | 0.55±0.05 | 0.47±0.05 | <0.001* | 0.68±0.06 | 0.64±0.06 | 0.012* |
| Stride Length (meters) | 1.32±0.17 | 1.33±0.16 | 0.815 | 1.62±0.19 | 1.63±0.18 | 0.835 | 0.87±0.32 | 1.01±0.30 | 0.086 | 1.39±0.17 | 1.41±0.17 | 0.65 | 1.41±0.12 | 1.42±0.11 | 0.738 | 1.02±0.18 | 0.94±0.18 | 0.091 | 1.04±0.10 | 0.93±0.14 | 0.001* | 1.31±0.11 | 1.29±0.11 | 0.484 |
